# An Increasing Triglyceride–Glucose Index Is Associated with a Pro-Inflammatory and Pro-Oxidant Phenotype

**DOI:** 10.3390/jcm13133941

**Published:** 2024-07-05

**Authors:** Beverley Adams-Huet, Ishwarlal Jialal

**Affiliations:** 1 UT Southwestern Medical Center, Dallas, TX 75235, USA; huet@digitrain.com; 2 UC Davis School of Medicine, 2616 Hepworth Drive, Davis, CA 95618, USA

**Keywords:** insulin resistance, triglyceride–glucose index, inflammation, metabolic syndrome

## Abstract

**Background/Objectives:** Insulin resistance is crucial in the pathogenesis of Metabolic Syndrome (MetS), type 2 diabetes mellitus (T2DM) and premature atherosclerotic cardiovascular disease (ASCVD). The triglyceride–glucose index (TyG index), a validated measure of insulin resistance, also predicts MetS, T2DM, the severity of albuminuria and ASCVD. There are scant data providing mechanistic insights into these sequalae. Accordingly, we investigated the relationship between the TyG index and biomarkers of inflammation, oxidative stress, free fatty acid (FFA) levels and adipokine dysregulation in a cohort comprising both controls and patients with nascent MetS. **Methods:** Participants (*n* = 102) included 59 patients with MetS and 43 controls. People with diabetes, ASCVD, smoking and macro-inflammation were excluded. Fasting blood was obtained for both plasma and monocyte isolation. **Results:** Receiver Operating Characteristic (ROC) curve analysis revealed that the TyG index was an excellent predictor of MetS with an area under the curve of 0.87, and it correlated with both hepatic and adipose tissue insulin resistance. Both serum RBP-4 levels and non-HDL cholesterol increased significantly over tertiles of the TyG index. Based on the TyG index tertiles and/or correlations, oxidized LDL, nitrotyrosine, C-reactive protein, endotoxin, chemerin, interleukin-6 levels and monocyte toll-like receptor (TLR)-4 and TLR-2 and their cellular signaling were significantly associated with the TyG index. **Conclusions:** Increased non-HDL-C and, most importantly, a pro-inflammatory and pro-oxidant state could be advanced as potential mechanisms explaining the increased risk for T2DM and ASCVD with an increasing TyG index.

## 1. Introduction

Insulin resistance predisposes individuals to Metabolic Syndrome (MetS), T2DM and ASCVD [1,2,3,4]. The triglyceride–glucose index (TyG) index, which has been validated against the hyperinsulinemic–euglycemic clamp technique, has been shown to be a reliable, cost-effective surrogate measure of insulin resistance [5,6,7,8,9,10,11]. The majority of these studies validating the TyG index emanate from Asian and Latin American populations [5,6,7,8,9,10,11]. 

In a recent report, we showed that the TyG index was superior to HOMA-IR for predicting MetS in a US adult population [12]. In addition to predicting MetS and diabetes, the TyG index also predicts cardiovascular diseases [13,14,15,16]. Furthermore, a recent report showed that it predicted the severity of albuminuria independent of insulin resistance and MetS [17]. In all of these disorders, both inflammation and oxidative stress have been suggested to be important pathogenic mechanisms [4,18]. However, there are scant data investigating a detailed repertoire of circulating and cellular biomarkers of inflammation and oxidative stress in people with increasing TyG indices [6,19]. In our recent report, in accordance with a study from Brazil [6], we showed that the TyG index correlated significantly with hsCRP levels [12].

Accordingly, in the present report, we investigate the relationship between the TyG index and biomarkers of inflammation, oxidative stress and adipokine dysregulation in a select cohort comprising both controls and nascent MetS using both tertiles of the TyG index and correlations with relevant variables.

## 2. Patients and Methods

In a series of papers, significant findings in this cohort focusing on adipokine dysregulation, inflammation and oxidative stress have been reported [20,21,22,23,24]. MetS participants (*n* = 59) and controls (*n* = 43) aged 21–75 years old were recruited from Sacramento County, CA, using the criteria of the Adult Treatment Panel III (ATP III), as described previously [1,2]. The five MetS risk factors included higher waist circumference (≥102 cm for men and ≥88 cm for women), elevated triglycerides (≥150 mg/dL), low HDL cholesterol levels (<40 mg/dL for men and <50 mg/dL for women), high blood pressure (systolic blood pressure ≥ 130 mmHg or diastolic blood pressure ≥85 mm Hg) and high glucose level (≥100 mg/dL). Participants were defined as having MetS if they had at least three cardio-metabolic features of MetS. The exclusion criteria for healthy control subjects included current use of any blood pressure medications, elevated triglyceride levels (>200 mg/dL) and having 3 or more of the ATP III criteria. Other important exclusion criteria for all subjects, which were determined via a screening questionnaire, clinical examination and baseline chemistries, included diabetes defined by a fasting blood glucose level > 125 mg/dL and HbA1C > 6.4%, clinical ASCVD, acute or chronic inflammatory disorders and a history of smoking [20,21,22,23,24]. Additionally, all participants in this study had a highly sensitive C-reactive protein (hsCRP) level < 10.0 mg/L and a normal white cell count. This study was first approved by the institutional review board at the University of California, Davis on 25 July 2007, and informed consent was obtained from all participants.

Fasting blood samples were taken from participants after histories and physical examinations. The details of the different assays have been reported previously [20,21,22,23,24]. Plasma lipids, lipoproteins and glucose were assayed via standard laboratory techniques in the Clinical Pathology Laboratory, as described previously [21,22]. Insulin levels were assayed via ELISA (Linco Biosystems, St. Charles, MO, USA), and a homeostasis model assessment insulin resistance index (HOMA-IR) was calculated from glucose and insulin levels according to the following formula: fasting insulin (mU/L) × fasting glucose (nmol/L)/22.5 [21]. Non-HDL was calculated as total cholesterol minus HDL cholesterol. Endotoxin levels were quantitated using reagents from Lonza (Limulus Amebocyte Lysate, QCL 1000; Walkersville, MD, USA). Levels of oxidized low-density lipoprotein (ox-LDL) and nitrotyrosine were measured in the plasma via sandwich ELISA using reagents from Mercodia (U.S. branch, Winston-Salem, NC, USA) and Bioxytech (Oxis Research International, Inc., Foster City, CA, USA), respectively. The surface expression of toll-like receptors (TLR2 and TLR4) on monocytes was analyzed via flow cytometry using the BD FACS Array, as reported previously [21]. Retinol binding protein 4 (RBP-4), chemerin, adiponectin and leptin levels were measured via ELISA using reagents from Linco. Interleukin-1 (IL-1), IL-6 and IL-8 were measured using a multiplex cytokine/chemokine array (Bioplex, San Jose, CA, USA). Nuclear factor Kappa-beta (NFkB) activity (phospho-p65 in nuclear extracts) and cytosolic phospho-P38 mitogen-activated protein (MAP) Kinase activity (pP38MAP Kinase) were assayed using the Bioplex multiplex phosphoprotein detection assay (Biorad) [21,24]. 

The following formula was used to calculate the Systemic Immune–Inflammation Index (SII index): SII = neutrophil count (10^9^/L) × platelet count (10^9^/L)/lymphocyte count (10^9^/L) [19].

The triglyceride–glucose (TyG) index was calculated as reported previously:

Ln [fasting triglycerides (mg/dL) × fasting plasma glucose (mg/dL)/2] [5,7].

Adipose tissue insulin resistance was calculated as the product of FFA and fasting insulin levels, as reported previously [25].

### Statistical Analysis

SAS version 9.4 (SAS Institute, Cary, NC, USA) was used for statistical analysis, and significance was defined as a two-sided *p*-value < 0.05. Results are expressed as the median and interquartile range. Trend analysis of the TyG index tertiles in our combined MetS and control participants was evaluated using the Jonckheere–Terpstra (J-T) test for trends. The Cochran–Armitage Trend Test was used to analyze categorical variables across tertiles of the TyG index. Combining the control and MetS groups, the Spearman rank correlation coefficients were also determined to assess the association between the TyG index and relevant variables. Age-adjusted partial correlation analysis was undertaken to control for possible confounding by age and did not alter the results. Logistic regression models were used to compute the Receiver Operating Characteristic (ROC) area under the curve (AUC) for assessing the efficacy of the TyG index, as the independent variable, in the prediction of MetS, the binary dependent variable. 

## 3. Results 

As depicted in Table 1, all cardio-metabolic features increased significantly with increasing tertiles of the TyG index, except for high-density lipoprotein cholesterol (HDL-C), which decreased significantly. In addition, fasting insulin, non-HDL cholesterol, FFA levels, hsCRP and HOMA-IR increased significantly with increasing tertiles of the TyG index. Importantly, a valid measure of adipose tissue insulin resistance (Adipo-IR) also increased significantly with increasing tertiles of the TyG index. The TyG index correlated significantly with both HOMA-IR (rho = 0.52, *p* < 0.0001) and Adipo-IR (rho = 0.68, *p* < 0.0001).

**Figure 1:**
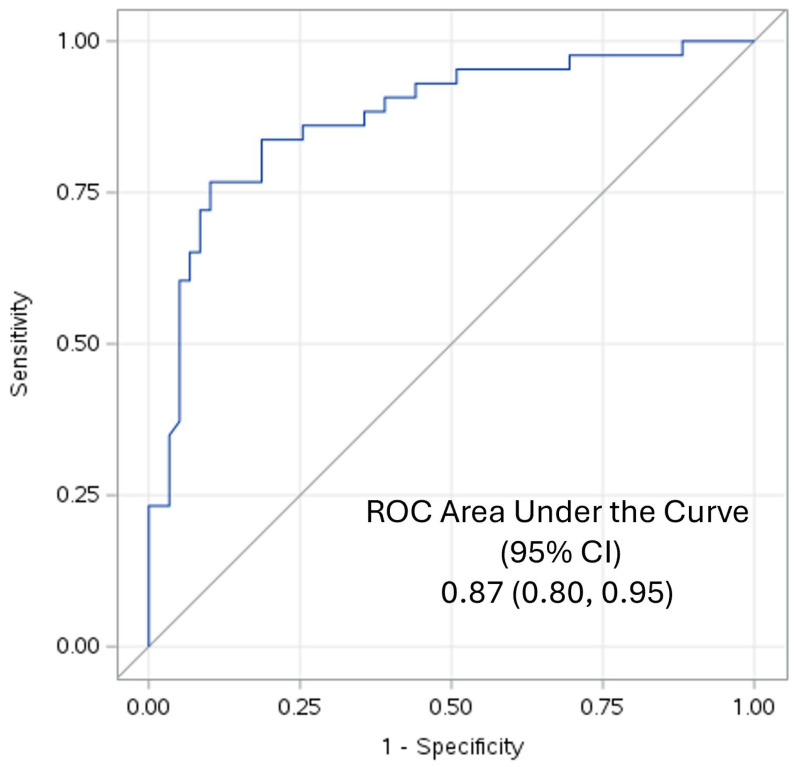
ROC-AUC of the TyG Index predicting MetS.

Since 59 participants had MetS and 43 were controls, ROC-AUC analyses were undertaken to determine the validity of the TyG index for predicting MetS. It revealed that the TyG index was an excellent predictor of MetS according to the criteria of Hosmer and Lemeshow [26]: it had an ROC-AUC of 0.87 with a 95% confidence interval of 0.80 to 0.95, as shown in Figure 1.

In previous reports, biomarkers of inflammation, oxidative stress and dysregulation of adipokine biology have been detailed in these participants [20,21,22,23,24,25]. In the present communication, the focus was on those biomarkers that were significantly abnormal in those published studies, concentrating on their relationships with increasing tertiles of the TyG index.

In Table 2 are shown various biomarkers of oxidative stress, inflammation and adipokines across tertiles of the TyG index. Oxidized LDL and plasma nitrotyrosine levels were significantly increased over the TyG index tertiles. With respect to circulating biomarkers of inflammation, in addition to the prototypic downstream marker of inflammation, hsCRP, endotoxin and IL-6 levels were increased significantly. However, none of the other cytokines, including IL-1 and IL8, were significantly increased over the TyG index tertiles.

### MFI-Mean Fluorescence Intensity

The two important toll-like receptors (TLR) relevant to diabetes and cardiovascular diseases are TLR2 and TLR4 [27]. Whilst TLR2 abundances on monocytes were not increased statistically, there was a significant increase in TLR4 abundances on monocytes with increasing tertiles of the TyG index. Furthermore, two important signal transduction pathways, nuclear factor Kappa-beta (NFkB) activity and cytosolic phospho-P38 mitogen-activated protein (MAP) Kinase activity (pP38MAP Kinase), were also increased in monocytes across tertiles. 

We examined four adipokines in this report. Serum chemerin and retinol binding protein 4 levels were significantly increased across the TyG index tertiles. Both leptin (*p* = 0.06) and adiponectin were non-significant over tertiles. 

We also undertook correlations to understand relationships with the TyG index given the paucity of data with respect to these biomarkers in the published literature. For all cardio-metabolic features presented in Table 1, the Spearman correlation coefficients paralleled the tertile analyses and were significant. 

In Table 3 is shown the correlations with the biomarkers reported in Table 2. The significant increases over tertiles of the TyG index were paralleled by significant correlations. In addition, monocyte TLR2 abundance and leptin levels revealed significant correlations. Age-adjusted partial correlations yielded similar results.

Since a previous study showed that the TyG index increased significantly over quartiles of the SII index [19], we also determined this relationship in our cohort. The median SII indices over tertiles were 425, 512 and 488, respectively, *p* for trend = 0.21 with a non-significant correlation; rho = 0.11, *p* = 0.29.

For simplicity and due to the paucity of data on the TyG index and these biomarkers, either significance defined by tertiles or correlations can be interpreted as significant for the discussion.

## 4. Discussion

The present report was prompted by the paucity of data on the relationship between the TyG index and biomarkers of inflammation and oxidative stress [6,19] to explain the increased risk for T2DM, ASCVD and the severity of albuminuria.

Our volunteers included controls and patients with nascent MetS without the confounding of T2DM, ASCVD, macro-inflammation, smoking and hypolipidemic drug therapy. Whilst the TyG index is a validated measure of insulin resistance, it also predicts MetS, diabetes, the severity of albuminuria and cardiovascular diseases [13,14,15,16,17]. Our ROC-AUC of 0.87 confirms that it is an excellent discriminant of MetS [12]. However, mechanistic insights to explain these associations are sparse. 

We make the novel observation that with increasing tertiles of the TyG index, there is a significant increase in a measure of adipose tissue insulin resistance, Adipo-IR. The strong correlation between the TyG index and Adipo-IR (r = 0.68) supports our previous hypothesis that it captures both hepatic and adipose tissue insulin resistance [12]. As expected, all cardio-metabolic features reported in Table 1 increased significantly, except for HDL-C, which decreased. The levels of non-HDL-C increased significantly with increasing tertiles of the TyG index with a correlation of 0.61 (*p* < 0.0001). This could also be advanced as a mediating mechanism for increased risk of cardiovascular diseases [28,29]. 

Oxidative stress is defined as an increase in reactive oxygen species and or reactive nitrogen species that exceeds the capacity of antioxidant defenses, resulting in footprints of injury to biomolecules [22,30]. With respect to oxidative stress as a mediating mechanism, our data on two biomarkers, oxidized LDL and nitrotyrosine, show that both increase with increasing tertiles of the TyG index, suggesting that oxidative stress could be advanced as a plausible mechanism for the increased risk of diabetes and ASCVD with increasing levels of the TyG index. Future studies need to focus on the relationship between the TyG index and biomarkers of oxidative stress to confirm these important preliminary findings.

A pro-inflammatory state is defined as an increase in biomediators of the inflammatory cascade, and this has recently been catalogued for MetS based on increases in multiple biomediators [31,32] In the present report, in addition to hsCRP and IL-6, endotoxin levels were significantly increased with the TyG index. Endotoxin is the classical ligand for the pattern recognition receptor TLR-4 [21]. FFA levels can contribute to the activation of TLR-4 and, thus, contribute to both insulin resistance and inflammation [4,33]. Furthermore, both cell surface receptors TLR-2 and TLR-4 displayed significant correlations with the TyG index. In addition, there were significant correlations with the downstream signal transduction pathways, especially for TLR-4, including the master switch of inflammation, NFKB activity and pP38 MAP Kinase activity. Thus, the activation of the TLR pathway could be a crucial mechanism linking the TyG index with inflammation and its adverse sequelae.

In summary, the above data on circulating biomarkers (increased hsCRP, endotoxin, IL-6 and chemerin levels) and cellular biomediators (increase in abundance of TLR2 and TLR4 and their signal transduction pathways) support the notion that a pro-inflammatory state is associated with an increasing TyG index.

The Systemic Immune–Inflammation (SII) Index has been shown to correlate with the TyG index. We failed to confirm this in our well-curated cohort controlling for confounders. The authors acknowledge that since this index is the product of neutrophils × platelets over lymphocytes, it is subject to confounding by comorbidities, drug therapy, etc. [19].

Adipokines are biomediators derived from adipose tissue that can ameliorate or enhance insulin resistance and inflammation, and adipokine dysregulation occurs when there are increases and/or decreases in adipokines with contrasting effects [31,34]. There is a significant increase in RBP-4 with increasing TyG index tertiles, and RBP-4 promotes insulin resistance [20]. Chemerin, a chemoattractant for macrophages and dendritic cells, is also an adipokine and appears to contribute to insulin resistance [35]. In a prospective study, chemerin predicted the onset of T2DM over a period of 5.3 years [36]. The increase in chemerin levels could also explain the relationship between the TyG index and the risk of diabetes. Unlike a report from Brazil [6], we found no significant correlation between plasma adiponectin levels and the TyG index. This could be explained by the different populations and assays used. There was a significant correlation between leptin levels and the TyG index. However, much further work is needed to establish links between the TyG index and adipokine dysregulation.

In conclusion, in participants without the confounding factors of T2DM, ASCVD, smoking, macro-inflammation and lipid therapy, the TyG index is an excellent predictor of MetS and captures both hepatic and adipose tissue insulin resistance. With respect to mechanistic insights, it appears, based on the above findings, that increased non-HDL-C (ASCVD risk), an increased RBP-4 (increased risk for diabetes) pro-oxidant state (increases in Ox-LDL and nitrotyrosine) and a pro-inflammatory state (evidenced collectively by increases in hsCRP, 1L-6, endotoxin, TLR-2 and TLR-4 abundance and activity and elevated chemerin levels) could be advanced as mediating mechanisms explaining the increased risk of T2DM and ASCVD associated with an increased TyG index. However, given the cross-sectional nature of this report, it cannot imply cause and effect. This can only be settled with prospective studies.

## Figures and Tables

**Table 1:** Cardio-metabolic features across tertiles of the TyG index in a cohort comprising both MetS and controls.

Variable	Tertile 1 *n* = 34	Tertile 2 *n* = 34	Tertile 3 *n* = 34	*p*-Value *
Female/Male, *n* (%)	27/7 (79/21)	27/7 (79/21)	24/10 (71/29)	0.39
Control/MetS, *n* (%)	29/5 (85/15)	11/23 (32/68)	3/31 (9/91)	<0.0001
TyG index	7.9 (7.7–8.2)	8.5 (8.4–8.6)	9.1 (8.9–9.3)	<0.0001
Age (years)	46 (41–56)	53 (45–61)	54 (48–60)	0.03
Waist (cm)	87 (81–99)	103 (95–117)	107 (97–117)	<0.0001
Weight (kg)	80.5 (71.4–91.8)	98.2 (84.1–111.4)	92.7 (82.7–112.3)	0.003
BMI (kg/m^2^)	29.0 (25.8–32.4)	34.3 (31.9–41.0)	33.8 (28.5–39.2)	0.002
Systolic BP (mmHg)	120 (108–132)	132 (116–139)	129 (122–136)	0.009
Diastolic BP (mmHg)	74 (66–82)	80 (72–86)	79 (75–86)	0.007
Glucose (mg/dL)	88 (83–95)	95 (88–102)	100 (94–109)	<0.0001
Insulin (mU/L)	6.0 (3.8–10.2)	10.6 (7.2–17.1)	13.0 (9.4–21.1)	<0.0001
Total cholesterol (mg/dL)	177 (159–198)	198 (183–213)	208 (188–224)	0.0008
HDL cholesterol (mg/dL)	54 (43–64)	47 (35–50)	35 (31–41)	<0.0001
Non-HDL cholesterol (mg/dL)	122 (108–147)	154 (136–161)	159 (152–181)	<0.0001
Triglycerides (mg/dL)	62 (50–70)	107 (97–122)	174 (156–211)	<0.0001
hsCRP (mg/L)	1.1 (0.4–4.0)	3.4 (1.3–5.4)	2.6 (1.7–4.6)	0.006
HOMA-IR	1.4 (0.8–2.3)	2.6 (1.6–3.7)	3.5 (2.2–5.7)	<0.0001
FFA (umol/L)	320 (180–440)	580 (400–760)	(780(670–950)	0.0001
Adipo-IR (mmol/pmol)	11 (6–36)	42 (27–69)	91 (55–106)	<0.0001

* The Jonckheere–Terpstra Test for trend for continuous variables and the Cochran–Armitage test for categorical variables. Adipo-IR—adipose tissue insulin resistance; FFA—free fatty acids. The results are reported as median values (25th–75th percentile).

**Table 2:** Biomarkers of oxidative stress, inflammation and adipokines across tertiles of the TyG Index in a cohort comprising both MetS and controls.

Variable	Tertile 1	Tertile 2	Tertile 3	* *p*-Value Jonckheere-Terpstra Test
**Oxidative stress:**				
Oxidized LDL (U/L)	25.4 (21.3–40.3)	43.9 (36.1–50.0)	45.2 (34.8–58.5)	0.0002
Nitrotyrosine (nM/L)	10.0 (5.9–24.2)	28.2 (10.9–64.2)	23.2 (13.7–102.9)	0.03
**Inflammation:**				
Endotoxin (EU/mL)	4.1 (3.5–4.7)	7.0 (3.7–10.6)	13.7 (11.5–17.7)	0.0004
IL-6 (pg/mL)	1326 (402–1775	1693 (565–2146)	1696 (1346–2018)	0.007
IL-8 (pg/mL)	768 (626–1113)	807 (588–1456)	897 (648–1488)	0.20
IL-1-beta (pg/mL)	818 (421–943)	844 (532–1187)	879 (563–987)	0.25
TLR-2 (MFI/10^6^ cells)	24 (18–31)	24 (21–51)	29 (20–45)	0.12
TLR-4 (MFI/10^6^ cells)	21 (19–29)	26 (21–29)	31 (25–54)	0.002
pP38MAP Kinase	0.07 (0.04–0.09)	0.14 (0.09–0.24)	0.23 (0.14–0.36)	<0.0001
NFkB activity	0.05 (0.04–0.07)	0.24 (0.07–0.27)	0.24 (0.12–0.26)	<0.0001
**Adipokines:**				
RBP4 (μg/mL)	38.2 (34.1–44.6)	44.6 (36.3–56.6)	48.9 (39.3–55.8)	0.009
Leptin (ng/mL)	35.0 (24.1–55.4)	67.4 (36.6–105.3)	65.9 (38.8–83.8)	0.06
Adiponectin (μg/mL)	7.8 (5.7–11.3)	5.6 (3.9–8.4)	5.1 (3.9–12.0)	0.10
Chemerin (ng/mL)	279 (234–333)	319 (273–387)	370 (311–408)	0.009

The results are reported as median values (25th–75th percentile). * The Jonckheere–Terpstra Test for trends.

**Table 3:** The Spearman rank correlations between the TyG index and biomarkers of inflammation, oxidative stress and adipokines in a cohort comprising both MetS and controls.

Variable	rho	*p*
**Oxidative stress**		
Oxidized LDL	0.57	**<0.0001**
Nitrotyrosine	0.30	**0.04**
**Inflammation**		
Endotoxin	0.64	**<0.0001**
Interleukin-6	0.28	**0.005**
Interleukin-8	0.15	0.16
Interleukin-1-beta	0.19	0.07
Monocyte–Toll-like receptor-2	0.23	**0.04**
Monocyte–Toll-like receptor-4	0.32	**0.003**
Monocyte–NFkB activity	0.43	**<0.0001**
Monocyte–pP38MAP Kinase activity	0.58	**<0.0001**
**Adipokines**		
Retinol binding protein 4	0.36	**0.001**
Leptin	0.24	**0.03**
Adiponectin	−0.13	0.26
Chemerin	0.42	**0.002**

## Data Availability

The data are available from the senior author for review upon reasonable request.
